# Inter-observer agreement among specialists in the diagnosis of Oral Potentially Malignant Disorders and Oral Cancer using Store-and-Forward technology

**DOI:** 10.21203/rs.3.rs-2754683/v1

**Published:** 2023-04-05

**Authors:** Keerthi Gurushanth, Nirza Mukhia, Sumsum P Sunny, Bofan Song, Shubhasini A Raghavan, Shubha Gurudath, Pramila Mendonca, Shaobai Li, Sanjana Patrick, Tsusennaro Imchen, Shirley T. Leivon, Tulika Shruti, Trupti Kolur, Vivek Shetty, Vidya Bhushan R, Rohan Michael Ramesh, Vijay Pillai, Kathryn O. S, Petra Wilder Smith, Amritha Suresh, Rongguang Liang, Praveen Birur N, Moni A. Kuriakose

**Affiliations:** KLE Society’s Institute of Dental Sciences; KLE Society’s Institute of Dental Sciences; Mazumdar Shaw Medical Foundation; University of Arizona; KLE Society’s Institute of Dental Sciences; KLE Society’s Institute of Dental Sciences; Mazumdar Shaw Medical Foundation; University of Arizona; Biocon Foundation; Christian Institute of Health Sciences and Research; Christian Institute of Health Sciences and Research; Homi Bhabha Cancer hospital; Mazumdar Shaw Medical Foundation; Mazumdar Shaw Medical Foundation; Mazumdar Shaw Medical Foundation; Christian Institute of Health Sciences and Research; Mazumdar Shaw Medical Foundation; Beckman Laser Institute, University of California Irvine School of Medicine; Beckman Laser Institute, University of California Irvine School of Medicine; Mazumdar Shaw Medical Foundation; University of Arizona; KLE Society’s Institute of Dental Sciences; Karkinos Healthcare

**Keywords:** Agreement, diagnosis, Oral cancer, potentially malignant, Specialist, Telemedicine

## Abstract

Oral Cancer is one of the most common causes of morbidity and mortality. Screening and mobile Health (mHealth) based approach facilitates remote early detection of Oral cancer in a resource-constrained settings. The emerging eHealth technology has aided specialist reach to rural areas enabling remote monitoring and triaging to downstage Oral cancer. Though the diagnostic accuracy of the remote specialist has been evaluated, there are no studies evaluating the consistency among the remote specialists, to the best of our knowledge. The purpose of the study was to evaluate the interobserver agreement between the specialists through telemedicine systems in real-world settings using store and forward technology. Two remote specialists independently diagnosed the clinical images from image repositories, and the diagnostic accuracy was compared with onsite specialist and histopathological diagnosis when available. Moderate agreement (k = 0.682) between two remote specialists and (k = 0.629) between the onsite specialist and two remote specialists in diagnosing oral lesions. The sensitivity and specificity of remote specialist 1 were 92.7% and 83.3%, whereas remote specialist 2 was 95.8% and 60%, respectively, compared to histopathology. The store and forward technology and telecare can be effective tools in triaging and surveillance of patients.

## Introduction

Cancer of the lip and oral cavity is the 16th most common cancer worldwide, with over 377,713 new incident cases and 177,757 deaths annually. India is the second country with the highest number of oral cancer cases ^[1]^. India accounts for about 100,000 incident cases accounting for nearly one-fourth of the overall burden, making oral cancer a leading cause of death among men ^[2, 3]^.

The stage of the disease at diagnosis is the prime determinant of the treatment outcome of the patients^[4]^. The 5-year survival rate for localized cancers is 54.3–60.2%, while it is as low as 3.1–3.3% in advanced stages^[5]^. Detection at an advanced stage lowers the chances of a cure, decreases the quality of life, and results in considerable cost to the patient ^[3]^. In India, 70% of the cases are reported in the advanced stages (American Joint Committee on Cancer, Stage III-IV), with five-year survival rates of around only 20%^[2]^. One important cause is the lack of access to an oral cancer specialist^[5]^. Moreover, early-stage cancers are often asymptomatic, decreasing the chances of the patients seeking the medical attention they need^[3]^.

Oral cancer is preceded by a group of visible mucosal lesions called Oral Potentially Malignant Disorders (OPMDs) that exhibit oral epithelial dysplasia^[6]^. Early detection and surveillance of such OPMDs have the potential of not only decreasing the incidence but also improving the survival of those who develop oral cancer. Conventional Screening provides an excellent opportunity for early detection and surveillance ^[3]^. Some of the drawbacks, however, are inefficient data management, poor follow-up after the screening, lack of knowledge and practice of dentists to recognize and diagnose OPMDs, and a breakdown or delay in communication with the specialist ^[7]^. Hence, the continuous challenge of effective specialist-patient communication and physical inaccessibility to healthcare services escalates the number of advanced-stage diagnoses and cancer mortality^[8]^.

A communication strategy such as telemedicine involving real-time technology, store-and-forward technology, remote monitoring, and mhealth approaches is reliable, acceptable, accessible, reduces the cost of travel, and unnecessary referral of the patients, can serve as an effective tool for triaging, and enhance the training for primary care physicians ^[4, 8, 9]^. In the present scenario, telemedicine systems are openly accepted by healthcare professionals in the health system in low-income countries^[8]^. Telemedicine program for remote diagnosis of OPMDs and Oral cancer screening has already been piloted in India. It was found that telemedicine-based oral cancer screening and surveillance were feasible in low-resource settings ^[4]^.

So far, most telemedicine systems studies on OPMDs and oral cancer conducted, assessed the agreement between the remote (single diagnosis) and the face-to-face/onsite diagnosis. The agreement and consistency among remote specialists have not been robustly assessed. The objective of this study was to determine the interobserver agreement between the onsite, two remote specialists, and histopathology and to evaluate the diagnostic accuracy of the remote specialists in a real-world setting. The study uses store-and-forward technology where the clinical photographs of these lesions taken by the Frontline Healthcare Providers (FHPs) were transferred digitally via a secure server for remote diagnosis and recommendations.

## Methodology

The prospective double-blinded study was conducted to evaluate the diagnostic efficacy of two remote specialists in diagnosing OPMDs and Oral cancer and also to determine the interobserver agreement of the two remote specialists in store and forward technology and mHealth telemedicine. The Institutional review board approved the study (KIDS/IEC/Nov-2018/18). The onsite specialist performed the conventional visual examination of the participants and provided a specific diagnosis. The lesions were biopsied based on an onsite specialist recommendation. The FHPs collected the clinical images using a smart-phone based mHealth application. The deidentified image data was stored in the image repositories of KLE Institute of Dental Sciences, Bengaluru. These clinical images (n = 822) accrued from the image repository were evaluated by two remote specialists. The remote diagnosis was performed based on the image data without information on associated risk factors such as age, gender, habit history, and underlying systemic disease/ drug history. The remote specialists were blinded and independently provided a diagnosis for each image under four categories as follows-

### Diagnostic criteria and diagnosis:

**Figure 1:**
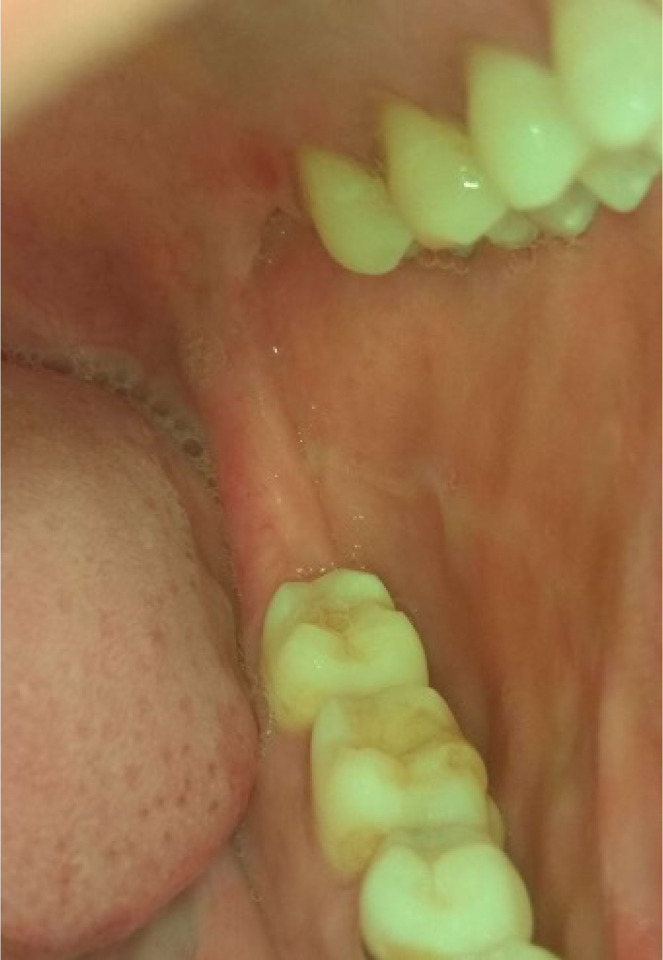
Photograph of normal buccal mucosa

**Figure 2:**
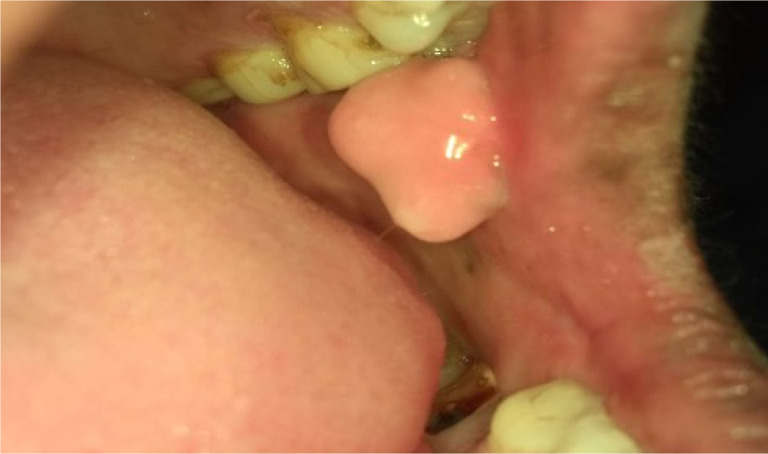
Photograph of benign exophytic growth on left buccal mucosa

**Figure 3:**
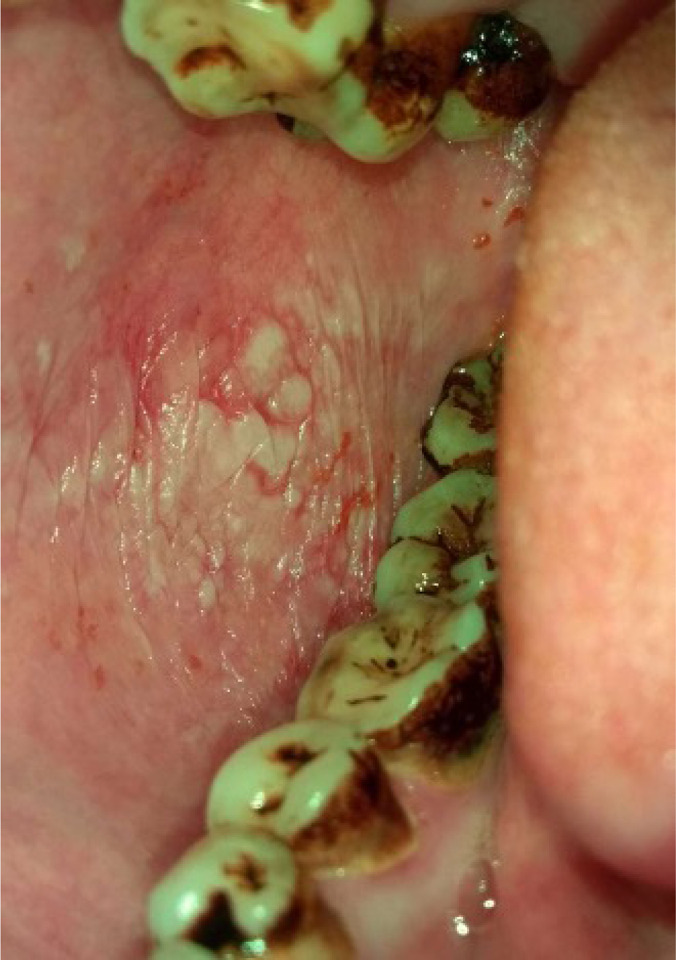
Photograph of Speckled leukoplakia (mixed red and white lesion) on right buccal mucosa

**Figure 4:**
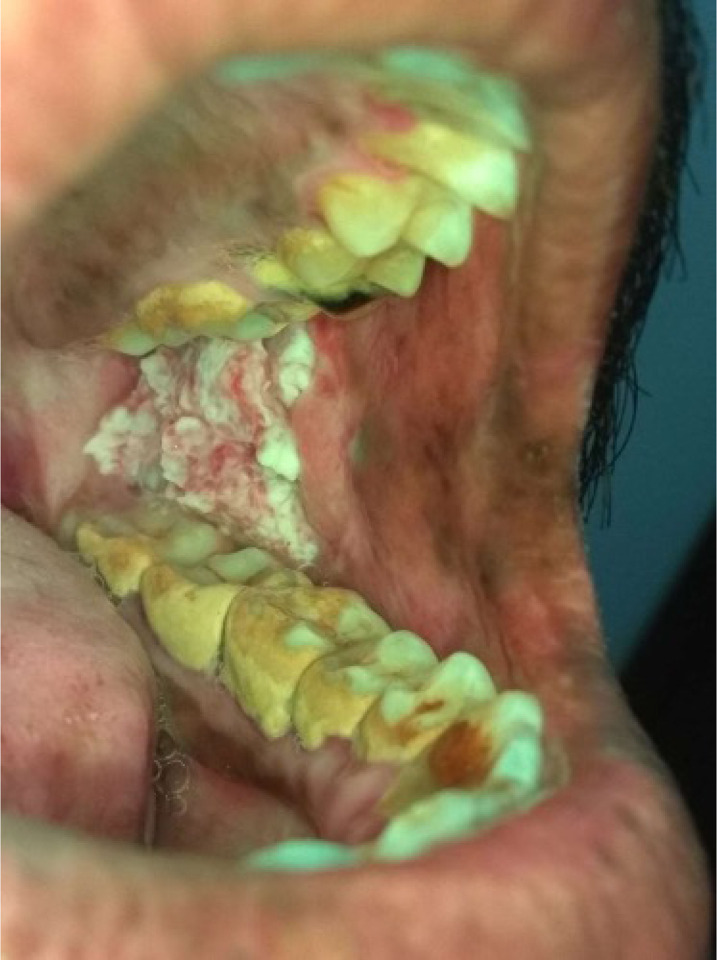
Photograph of malignant proliferative growth on left buccal mucosa

1. *Category 1* – Normal/ normal variations (Fig. 1)
2. *Category 2* - Benign (fibroma, inflammatory hyperplasia’s, smoker’s melanosis, paan encrustations, paan stains, [Paan is the mixture of betel leaf, nut and slake lime with or without tobacco] and frictional keratosis etc). (Fig. 2)
3. *Category 3*- Oral Potentially malignant disorders (Homogenous leukoplakia, nonhomogeneous leukoplakia, Oral lichen planus, Oral Submucous Fibrosis (OSMF), Tobacco pouch keratosis) (Fig. 3)
4. *Category 4*- Malignancy (Fig. 4)

The diagnostic accuracy of the remote specialists was compared with the onsite specialist, who we have considered as the reference standard.

A total of (n = 102) biopsies were performed based on onsite specialist recommendation, which was considered the gold standard. The two onsite, remote specialists’ diagnosis was compared with histopathology. The diagnostic accuracy in terms of sensitivity, specificity, positive predictive, and negative predictive values were determined, and the interobserver agreement was estimated using Cohen’s kappa. Cohen suggested the Kappa result be interpreted as follows: values ≤ 0 as indicating no agreement and 0.01–0.20 as none to slight, 0.21–0.40 as fair, 0.41– 0.60 as moderate, 0.61–0.80 as substantial, and 0.81–1.00 as almost perfect agreement ^[10]^.

## Results

A total of (n = 822) images were included in the study to evaluate the interobserver agreement between the two remote specialists. Based on the onsite specialist diagnosis who is considered the reference standard, category 1 included (n = 228), category 2 (n = 147), category 3 (n = 326), and category 4 (n = 121) images.

### Concordance between the two remote specialists and the Onsite specialist-

Table 1 shows a substantial agreement (k = 0.682) between the two remote specialists in the diagnosis of clinical images. A moderate agreement (k = 0.603) was observed between the onsite specialist and remote specialist 1 in diagnosing oral lesions (Table 2) with sensitivity and specificity of 83.1% and 90.6% respectively. The sensitivity and specificity between the onsite specialist and remote specialist 2 was 79% and 82.6% respectively with moderate agreement (k = 0.605) in diagnosis of oral lesions as shown in Table 3. Table 4 shows the concordance between the onsite specialist and the two remote specialists in diagnosing oral lesions with a moderate agreement (k = 0.629). The diagnostic accuracy and concordance between the onsite specialist, remote Specialist 1, remote Specialist 2 are presented in Table 5.

### Concordance between the Histopathology, two remote specialists, and Onsite specialist-

A total of (n = 102) biopsies were obtained to compare the efficacy of the onsite specialist, remote specialist 1, and 2 with histopathology (the gold standard). Table 6 lists the diagnostic accuracy and concordance between histopathology, onsite, remote 1, and 2. The onsite specialist showed a sensitivity of 94.8% and specificity of 83.3% for diagnosing OPMDs and oral cancer with histopathology as the gold standard. remote specialist 1 showed sensitivity and specificity of 92.7% and 83.3%, in comparison, remote specialist 2 showed 95.8% sensitivity but a lower specificity of 60%.

## Discussion

A sustainable technology-based practice in healthcare is ever impressive. One such concept of ideal technology is telemedicine. Not long ago, the global pandemic crisis highlighted the need for telecommunication technology for communication strategies between the doctor and the patient, which has further addressed the need to evaluate existing facilities and the reliability of telehealth in reducing cancer-specific morbidity and mortality. The main aim of telehealth technology is to provide access to health care in rural areas where the need for the early diagnosis of OPMDs and oral cancer is high but has limited access to healthcare.

The mHealth–based remote oral cancer surveillance program adopted to aid in remote early detection of oral cancer by primary care dental practitioners in a resource-constrained setting has already been demonstrated ^[5]^. Store and forward technology, could eliminate the need for an onsite consultation in 50% of cases. In two isolated studies, the agreement between the onsite and the remote specialist was 97% and 92.7% in identifying oral lesions. Following this, the remote specialists formulated the diagnosis and recommendation for referrals ^[5,9]^. In another study, the remote specialists showed a sensitivity and specificity of 95% and 84% in diagnosing OPMD and oral cancer, respectively, when compared to the onsite specialist ^[11]^.

In our study, we evaluated the accuracy and interobserver agreement between remote specialists involved in a store-and-forward technology and mHealth telemedicine technology. The remote diagnosis used images obtained by low-skilled FHWs in low-income and limited infrastructure settings. The data collected by the FHWs included patient demographics, medical history, habit history, and photographs of the oral lesions. The remote diagnosis was done based on the morphology of the lesions (unstructured data) without any clinical data or patient risk factors, to maintain the uniformity of the data input for both machine learning and remote specialist. However, in regular telemedicine module, the remote specialist has an information on structured data (patient demographic details that includes the de-identified data, and habit history) and unstructured data (clinical images).

A study compared the diagnostic accuracy of remote versus onsite specialist visits using a novel, low-cost telehealth platform consisting of a smartphone-based, remote intraoral camera and custom software application found that on-site diagnosis showed high sensitivity (94%) and 69.2% specificity when compared with histopathological diagnosis, which did not significantly differ from the accuracy of remote specialist (sensitivity: 94%; specificity: 62.5%) ^[12]^. Our study showed a specificity of 83.3% by the remote specialist 1, but a 60% specificity by the remote specialist 2, the reason being more years of experience in telediagnosis for the former as compared to the latter.

The variability in the diagnosis provided by the remote specialists may also be due to misdiagnosis or overdiagnosis. The predefined clinical data and images of the lesions collected at the remote location might not be sufficient for the remote specialist to arrive at a diagnosis. In such a scenario, a diagnosis may be given based on perception and intuition rather than analytical thought, a human cognitive factor, which might enhance the incidence of misdiagnoses ^[13]^. A lesion with typical clinical features such as oral leukoplakia or oral lichen planus is easier to identify in an image than a case of oral submucous fibrosis and other malignant lesions (such as salivary gland tumors), which requires a thorough physical examination by the onsite specialist. The low specificity of the remote specialist, when compared to the histologic confirmation, may be due to the overdiagnosis of the lesions. The remote specialists were calibrated to over-diagnose in regular workflow, and the remote specialist diagnosis was provisional. Without the usual inspection and palpation of the lesion, the remote diagnosis relies on the clinical data and morphology of the lesions. It has been shown that early borderline lesions may be categorized as malignant to avoid the consequences of the misdiagnosis of more aggressive cancers ^[14]^. Though not all misdiagnoses result in harm, in reality, the malignant transformation of OPMDs and the aggressiveness of OSCCs is highly variable and unpredictable, and the relative contribution of overdiagnosis bias remains to be elucidated across populations ^[15]^.

Assessing and addressing concerns with respect to mHealth and store and forward telemedicine is a critical step towards fully integrating telemedicine in everyday clinical and outreach programs. All of this work is done in collaboration with community healthcare workers and general dentists. The patients, specialist of the practitioner need not be available at the same time, hence this model is convenient and improves efficiency, reduces travel and waiting time of patients, second opinions quickly obtained, and specialists report quickly obtained are some of the benefits of the store and forward technology. Limitations, such as the requirement of a good internet connection with both download and upload speeds at the remote location, overdiagnosis by the specialist, and privacy and security: protecting individual health records pose significant challenges ^[16]^. The diagnosis of the remote specialists depends on the quality of the photographs captured and the data collected from the remote location. The oral cavity is more difficult to diagnose in a teleconsultation because they are more difficult to photograph. FHPs should be trained in identifying the lesion and ensure proper focus on the lesion to get sharper images for diagnosis ^[9]^. When the quality of the image is nondiagnosable, smartphones with AI-driven applications may be deployed to alert the FHWs to retake the photograph. In this study, the FHWs were intimated by the remote specialists to retake the photograph. Store and forward technology is an asynchronous service model that relies on data and information sharing outside of real-time consultations.

## Conclusion

Store and forward technology can be an effective tool in triaging patients, surveillance and can strengthen the healthcare system in low- and middle-income countries. Training of remote specialists is recommended for telediagnosis to improve their efficiency. Despite limitations, telemedicine allows specialized clinicians to reach out to a larger number of cases than the geographic distance would permit. It also improves the process by avoiding delays in diagnosing oral cancer. The image repository also serves as an important tool for documenting visual changes over time.

## Figures and Tables

**Table 1:** Concordance between the two remote specialists

Remote 1	Remote 2				
	Category 1	Category 2	Category 3	Category 4	Total
Category 1	144	22	22	0	188
Category 2	17	82	29	4	132
Category 3	24	30	303	18	375
Category 4	0	9	7	111	127
Total	185	143	361	133	822

CI- Confidence Interval

**Table 2:** Concordance between the onsite specialist and remote specialist 1-

Onsite	Remote 1				
	Category 1	Category 2	Category 3	Category 4	Total
Category 1	149	6	70	3	228
Category 2	23	112	2	10	147
Category 3	12	13	259	42	326
Category 4	4	1	44	72	121
Total	188	132	375	127	822

CI- Confidence Interval

**Table 3:** Concordance between the onsite specialist and remote specialist 2-

Onsite			Remote 2		
	Category 1	Category 2	Category 3	Category 4	Total
Category 1	154	2	72	0	228
Category 2	13	102	18	14	147
Category 3	18	37	260	11	326
Category 4	0	2	11	108	121
Total	185	143	361	133	822

Cohen’s Kappa statistic (k) = 0.605 (CI-95%, 0.618–0.702)

Strength of agreement- Moderate

CI- Confidence Interval

**Table 4:** Concordance between the onsite specialist and two remote specialists-

Rating Category	Conditional Probability	Kappa	P-value	Fleiss Kappa
Category 1	0.244	0.661	0.00	0.629
Category 2	0.171	0.640	0.00	Moderate
Category 3	0.431	0.603	0.00	
Category 4	0.155	0.621	0.00	

**Table 5:** Diagnostic accuracy and concordance between onsite specialist and two remote specialists-

	Onsite Specialist Vs Remote Specialist 1	Onsite Specialist Vs Remote Specialist 2
True positive	417	390
True negative	290	271
False positive	30	57
False negative	85	104
Sensitivity	83.1%	79%
Specificity	90.6%	82.6%
PPV	91.3%	84.4%
NPV	81.8%	76.8%
Cohens Kappa (CI 95%, p = 0.001)	0.715	0.601

**Table 6:** Diagnostic accuracy and diagnostic concordance between histopathology, onsite remote specialist 1 and 2

	Histopathology Vs Onsite Specialist	Histopathology Vs Remote Specialist 1	Histopathology Vs Remote Specialist 2
True positive	91	89	93
True negative	05	05	03
False positive	01	01	02
False negative	05	07	04
Sensitivity	94.8%	92.7%	95.8%
Specificity	83.3%	83.3%	60%
PPV	98.9%	98.9%	96.1%
NPV	50%	41.7%	58.8%
Cohens Kappa (CI 95%, p = 0.001)	0.519	0.419	0.47
